# Agreement and Accuracy of the Dural Tail Sign for Differentiating Canine Meningioma From Glioma on MRI

**DOI:** 10.1111/vru.70169

**Published:** 2026-04-20

**Authors:** John F. Griffin, Alexander R. Briggs, Samantha Glamann, Nick D. Jeffery, Jonathan M. Levine, Andra K. Voges, Joseph M. Mankin, Sharon C. Kerwin, Clifford R. Berry, Wilfried Mai

**Affiliations:** ^1^ Department of Large Animal Clinical Sciences Texas A&M University College Station Texas USA; ^2^ Department of Small Animal Clinical Sciences Texas A&M University College Station Texas USA; ^3^ Department of Clinical Sciences Auburn University Auburn Alabama USA; ^4^ Department of Clinical Sciences and Advanced Medicine, Section of Radiology University of Pennsylvania School of Veterinary Medicine Philadelphia Pennsylvania USA

**Keywords:** central nervous system, CNS, magnetic resonance imaging, neoplasia, tumor

## Abstract

The dural tail sign (DTS) is an imaging sign used to categorize intracranial masses based on magnetic resonance imaging (MRI) axial localization. It is common in masses arising from outside the neuroparenchyma (extra‐axial) and rare in masses arising from the neuroparenchyma (intra‐axial). Accuracy and agreement reports of the DTS in canine meningioma are variable. Therefore, our objectives were to report agreement, sensitivity, and specificity of the DTS in dogs with a known diagnosis of meningioma or peripherally located glioma. This was a retrospective, secondary analysis, and cross‐sectional study. Dogs with histologically confirmed meningioma or peripherally located glioma and 3T MRI were included. A total of 27 cases, 16 meningioma and 11 glioma, were included. Post‐contrast T1‐weighted images were provided to five blinded image evaluators in two separate randomized sessions separated by a 6‐week washout period. Evaluators were asked to rate cases as “positive,” “negative,” or “indeterminate” for the DTS. All dogs with meningioma were rated positive for the DTS by a majority of evaluators in each session. The sensitivity and specificity for the DTS for the first session were 95% and 89.1%, respectively. Interobserver agreement for identifying the DTS was substantial (*κ* = 0.74), and intraobserver agreement across the two sessions was almost perfect (*κ* = 0.84). These findings indicate that, whereas not perfect, the DTS is accurate and reliable in the MRI differentiation of canine meningioma from glioma.

## 1 Introduction

Meningiomas and gliomas account for approximately 80%–85% of canine primary intracranial neoplasia [1, 2]. Accurate magnetic resonance imaging (MRI) diagnosis is critical yet challenging, particularly in distinguishing meningiomas from gliomas that contact the pial surface of the brain. For example, the reported sensitivity and specificity of MRI for the diagnosis of meningioma are ∼65% and ∼94%, respectively [3]. These challenges have been addressed by several studies to determine the accuracy and agreement of various imaging features (signs), novel acquisition techniques (e.g., diffusion/perfusion imaging), and novel image analysis techniques (e.g., radiomics and machine learning) [4, 5, 6, 7, 8, 9, 10, 11, 12, 13, 14].

The dural tail sign (DTS) was first reported in post‐contrast T1‐weighted images of human meningioma in 1989 and canine meningioma in 1996 [15, 16]. The DTS is defined as a linear enhancement of thickened dura adjacent to an extra‐axial mass that is continuous with the mass, contrast‐enhanced to an equal or greater degree than the mass, and seen in at least two contiguous slices or in two imaging planes [17, 18]. Although the DTS is most commonly associated with meningioma in dogs, it has also been reported in intracranial histiocytic sarcoma, blastomycosis, leptomeningeal oligodendrogliomatosis, and pituitary macroadenoma, as well as multiple conditions in people [19, 20, 21, 22, 23, 24]. The DTS in people is caused by a neoplastic infiltrate in ∼72% of cases, whereas nonneoplastic processes (e.g., vascular changes, connective tissue proliferation, and inflammation) account for the remainder [25, 26]. Importantly, neoplastic tissue in nearby non‐enhancing dura has been reported in up to 40% of human cases, underscoring the limitations of MRI and the DTS in defining the extent of neoplastic tissue [25, 26]. Reports of MRI/histopathological correlation of the DTS in dogs are lacking.

Estimates of the prevalence of DTS in canine meningioma range between 23% and 100% [15, 18, 20, 27, 28, 29]. Reports of agreement are similarly variable. For example, two evaluators disagreed on the presence or absence of the DTS in four out of 10 dogs in one report and one out of 11 dogs in another [18, 28]. One study reported a range of interobserver agreement (using the kappa statistic) from 0.22 to 0.74 amongst 5 veterinary radiologists [4]. This study did not analyze intraobserver agreement or the accuracy of any specific sign for the diagnosis of meningioma. The cause of the wide range in DTS prevalence and agreement is unknown, but differences in DTS definition, imaging protocols, population selection, and evaluator training and background may contribute. Therefore, our objectives were to report intra and interobserver agreement, sensitivity, and specificity of the DTS in a population of dogs with a known diagnosis of meningioma or peripherally located glioma. Our secondary objective was to review cases of disagreement for any discernible patterns.

## 2 Materials and Methods

This was a retrospective, secondary analysis, and cross‐sectional study design. This study was performed concurrently with another project aiming to report the agreement and accuracy of the claw sign [5]. The present study utilized the same population of dogs and a different set of image evaluators. The hospital director approved the use of the images for the study. Client consent was obtained for all dogs as part of the admission form. An approved protocol by the Institutional Animal Care & Use Committee was not required due to the retrospective nature of the study. All dogs that received a brain MRI (3 T) at the Texas A&M University College of Veterinary Medicine & Biomedical Sciences Small Animal Hospital between 2011 and 2021 and had a confirmed histopathologic diagnosis of meningioma or glioma on biopsy or necropsy were eligible for inclusion in the study. Cases were required to have post‐contrast, three‐dimensional T1‐weighted gradient‐echo images. Cases with glioma were evaluated for inclusion/exclusion by an investigator (JFG) who was not involved in the image analysis for agreement and accuracy. Only dogs with gliomas with pial contact were included, as those that do not contact the pia are unlikely to be confused with meningiomas. In patients who had multiple MRI studies, only the initial study was used.

Anonymized and randomized MRI studies were evaluated by three board‐certified radiologists (A.K.V., C.R.B., and W.M.) and two board‐certified neurologists (J.M.M. and S.C.K.), all of whom were experienced interpreters of canine brain MRI. Evaluators were not given case demographic information (e.g., breed and age). They used the DICOM viewer of their choice and evaluated only T1‐weighted post‐contrast images in the transverse, sagittal, and dorsal planes. If all planes were not available, evaluators were instructed to use multiplanar reconstruction to create them from the unreconstructed 3‐dimensional data set. A training set of 10 cases and videos was provided to image evaluators in advance. The training case population was similar to the study population, but cases had a presumptive clinical diagnosis without histopathological confirmation. Evaluators were provided with a key and explanatory comments regarding the training set. None of the evaluators asked any questions about the training cases. Evaluators rated each case as positive, negative, or indeterminate for the DTS. The DTS was defined as a linear enhancement of thickened dura adjacent to and continuous with a mass, which contrast‐enhanced to an equal or greater degree than the mass and was seen in at least two contiguous slices or in two imaging planes. The “indeterminate” category was assigned to a case when an evaluator could not categorize it as positive or negative. After the first image evaluation session, there was a 6‐week washout period before repeating the evaluation in a different randomized viewing order. Thus, each case was evaluated a total of 10 times (five evaluators and two sessions).

Power analysis for sample size determination was not performed; the sample size was determined by convenience. Evaluator responses were reported in a table. Cases of disagreement were reviewed by the first author to identify patterns. Statistical methods were selected and performed by one coauthor (N.D.J.) with extensive training and experience in statistical analysis using STATA 17 (StataCorp, College Station, TX, USA). Data were dichotomized as “positive” and “not positive.” “Not positive” included negative and indeterminate results. Cohen's and Fleiss's kappa statistic and 95% confidence intervals (CIs) were used to determine intra and interobserver agreement and variability. Kappa statistics were interpreted according to a common 5‐tiered convention [30]. Sensitivity and specificity (and their 95% CIs) were calculated for each evaluator for the first viewing session. A true positive was “positive” for the DTS and had a diagnosis of meningioma. A true negative was “not positive” for the DTS and had a diagnosis of glioma. A false positive was “positive” for the DTS and had a diagnosis of glioma. A false negative was “not positive” for the DTS and had a diagnosis of meningioma.

## 3 Results

The study population included 27 dogs, 16 with meningioma and 11 with glioma. The meningioma group included six neutered males and 10 spayed females. In the glioma group, there were five neutered males, two intact males, and four spayed females. Breeds were Golden Retriever (*n* = 2), Weimaraner (*n* = 1), German Shepherd dog (*n* = 2), Akita (*n* = 1), Shih Tzu (*n* = 1), Australian Shepherd dog (*n* = 1), Labrador Retriever (*n* = 3), Havanese (*n* = 1), mixed breed (*n* = 2), Chihuahua (*n* = 1), Dachshund (*n* = 1), Boxer (*n* = 6), French Bulldog (*n* = 3), Boston Terrier (*n* = 1), and American Pit Bull Terrier (*n* = 1). Dogs ranged from 4 to 15 years of age (median, 10 years). Dogs ranged from 3 to 54 kg body weight (median, 27 kg).

Post‐contrast, 3‐dimensional, T1‐weighed gradient echo images (magnetization‐prepared rapid gradient echo, MP‐RAGE) obtained on a 3 T MRI system (Magnetom Verio, Siemens Medical, Malvern, PA) were used for this study. Repetition time (TR) was 2100 ms, and echo time (TE) was 3–3.5 ms. Slice thickness ranged from 0.5 to 2.5 mm with no interslice gap. Matrix size was most commonly 256 × 256. Gadolinium dimeglumine (Magnevist, Bayer Healthcare Pharmaceuticals, Wayne, NJ) was used for 20 cases at 0.11 mmol/kg. Gadobutrol (Gadavist, Bayer Healthcare Pharmaceuticals, Wayne, NJ) was used in four cases at 0.22 mol/kg. All four dogs receiving the higher dose of Gadobutrol were in the glioma group, and all had perfect negative agreement for the DTS. The type and dose of contrast medium were not recorded in the medical records of three dogs. The time delay between contrast medium injection and imaging was not recorded.

**FIGURE 1:**
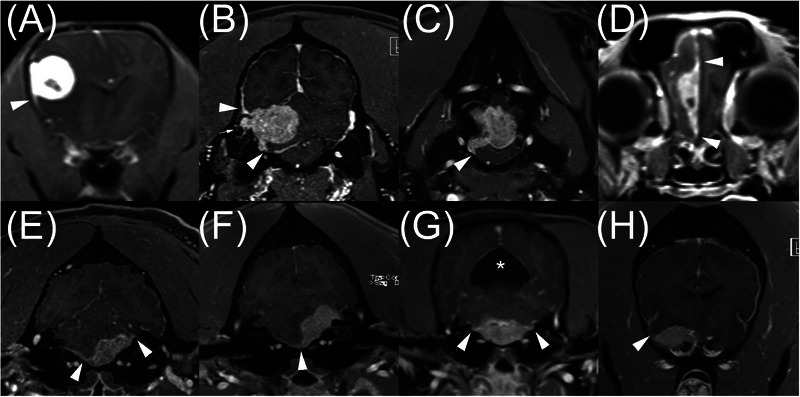
Post‐contrast, three‐dimensional, T1‐weighed gradient echo images of eight dogs (A‐H) with meningioma. These cases had perfect positive agreement for the dural tail sign (DTS, white arrowheads). The small white arrow in (B) denotes erosion of the neurocranium, and the asterisk in (G) denotes a supracollicular fluid accumulation. Slice thickness was 2.5 mm in all images.

**FIGURE 2:**
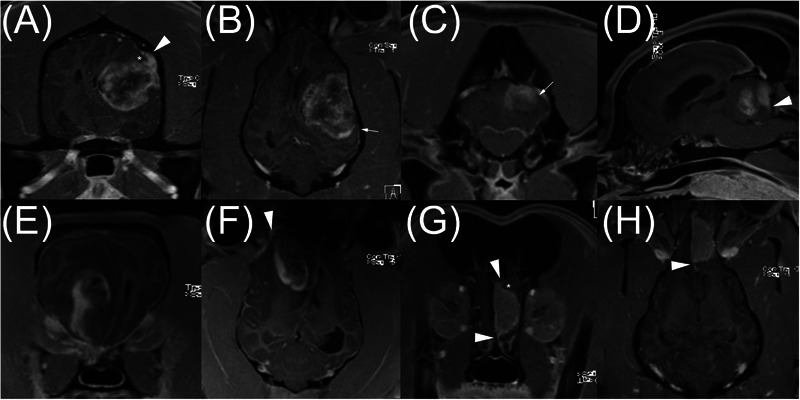
Post‐contrast, three‐dimensional, T1‐weighed gradient echo images of a boxer with a glioma (A and B), a French Bulldog with glioma (C and D), a Boston Terrier with glioma (E and F), and a Weimaraner with meningioma (G and H). There was disagreement regarding the dural tail sign (DTS) in five, four, three, and three out of 10 instances, respectively. The white arrowheads denote the DTS, and the small white arrows in B and C denote the claw sign. The boxer (A and B) and the Boston terrier (E and F) are examples of cases in which it is possible that some evaluators under‐read the DTS because they were biased by other imaging features (e.g., expansion of brain parenchyma and claw sign). In the French Bulldog (C and D), the DTS was not clearly seen on adjacent slices and orthogonal planes. The reason for disagreement in the Weimaraner (G and H) was unclear. In A and G, the asterisks denote thinning of brain parenchyma adjacent to the mass at the pial surface of the brain, forming an acute angle with a sharp point. These do not meet the criteria for the claw sign, as the vertex of the angle is displaced inwardly by the DTS. Slice thickness was 2.5 mm in all images.

Evaluators’ responses are included in Table 1. Sensitivity of the DTS for diagnosis of meningioma was 95% (95% CI, 87.7–98.6). Specificity was 89.1% (95% CI, 77.8–95.9). All dogs with meningioma were rated positive for the DTS by a majority of evaluators in each session. Examples of cases with perfect positive agreement are shown in Figure 1. The four cases with the worst agreement are shown in Figure 2. In the 11 dogs with glioma, there were eight cases of perfect negative agreement for the DTS and three cases of disagreement. These three dogs were rated positive for the DTS three, four, and five times out of the 10 evaluations for each dog. In the 16 dogs with meningioma, there were 11 cases of perfect positive agreement for the DTS and five cases of disagreement. These five dogs were rated as “not positive” for the DTS one, one, one, two, and three times out of 10.

**TABLE 1:** Evaluators’ responses.

Dog	Evaluator 1	Evaluator 1	Evaluator 2	Evaluator 2	Evaluator 3	Evaluator 3	Evaluator 4	Evaluator 4	Evaluator 5	Evaluator 5	Summary
	S1	S2	S1	S2	S1	S2	S1	S2	S1	S2	
G‐1	N	N	N	N	I	N	N	N	N	N	PNA
G‐2	N	N	N	N	N	N	N	N	N	N	PNA
G‐3	N	N	N	N	N	N	N	N	N	N	PNA
G‐4	N	N	N	N	N	N	N	N	N	N	PNA
G‐5	N	N	N	N	N	N	N	N	N	N	PNA
G‐6	N	N	N	N	N	N	N	N	N	N	PNA
G‐7	N	N	N	N	N	N	N	N	N	N	PNA
G‐8	N	N	N	N	N	N	N	N	N	N	PNA
G‐9	N	N	P	P	P	P	N	P	N	N	D
G‐10	P	P	N	N	P	P	N	N	N	N	D
G‐11	N	N	N	N	P	I	P	N	N	P	D
M‐1	P	P	P	P	P	P	P	P	P	P	PPA
M‐2	P	P	P	P	P	P	P	P	P	P	PPA
M‐3	P	P	P	P	P	P	P	P	P	P	PPA
M‐4	P	P	P	P	P	P	P	P	P	P	PPA
M‐5	P	P	P	P	P	P	P	P	P	P	PPA
M‐6	P	P	P	P	P	P	P	P	P	P	PPA
M‐7	P	P	P	P	P	P	P	P	P	P	PPA
M‐8	P	P	P	P	P	P	P	P	P	P	PPA
M‐9	P	P	P	P	P	P	P	P	P	P	PPA
M‐10	P	P	P	P	P	P	P	P	P	P	PPA
M‐11	P	P	P	P	P	P	P	P	P	P	PPA
M‐12	N	P	P	P	P	P	P	N	P	N	D
M‐13	P	P	P	P	P	I	P	P	P	P	D
M‐14	N	P	P	P	P	P	P	P	P	P	D
M‐15	I	P	P	P	P	P	P	P	P	P	D
M‐16	N	N	P	P	P	P	P	P	P	P	D

*Note*: Sensitivity and specificity were calculated from the first viewing session (S1). Responses were dichotomized so that both N and I were considered “not positive.” G1–11 are the dogs with glioma; M1–16 are the dogs with meningioma.

Abbreviations: D, disagreement; I, indeterminate; N, negative; P, positive; PNA, perfect negative agreement; PPA, perfect positive agreement; S1, session 1; S2, session 2.

The primary author reviewed the eight cases of disagreement with knowledge of the final diagnosis in order to identify patterns. In at least two dogs (one meningioma and one glioma), it seemed that the problem was related to the criterion that the DTS be seen in at least two contiguous slices or in two imaging planes. It was reasonably convincing in one image but equivocal in the other. Both of these dogs had a 2.5 mm slice thickness. In the three dogs with glioma, there were also other features (e.g., expansion of brain parenchyma rather than compression and low degree of contrast enhancement) that may have biased evaluators away from the DTS. In the remaining dogs, the reasons for disagreement were unclear. Of the dogs with disagreement, all five of the meningioma cases and one out of the three glioma cases involved the olfactory region.

Cohen's kappa statistic for intraobserver agreement for the DTS was almost perfect at 0.84 (95% CI, 0.75–0.94). Fleiss's kappa statistic for interobserver agreement was substantial at 0.74 (95% CI, 0.55–0.92).

## 4 Discussion

In this population of dogs with meningiomas or peripherally located gliomas with pial contact, the DTS was highly sensitive (95%) and reasonably specific (89.1%) for the diagnosis of meningioma. All dogs with meningioma had a DTS recognized by the majority of evaluators in each session. There was perfect positive or negative agreement in 19 out of 27 dogs.

The DTS compares favorably to the claw sign, which was 85.5% sensitive (95% CI, 73.3–93.5) and 80% specific (95% CI, 69.6–88.1) for the differentiation of superficially located glioma with pial contact from meningioma in the same population [5]. The claw sign is defined as thinning of brain parenchyma adjacent to a mass at the pial surface of the brain, forming an acute angle with a sharp point, the vertex of which is not displaced inwardly from the neurocranium, falx cerebri, or tentorium cerebelli [5]. Similar to the DTS, the claw sign must be seen on at least two contiguous slices or in two planes [5]. Disagreement for both the DTS and claw sign tended to be more common in the olfactory region [5]. The reasons for this are uncertain, but it may be related to the relatively large size of these masses relative to the small size of the rostral neurocranium.

The DTS and claw sign are not the exact inverse of each other, but they do interact with each other. For example, the linear meningeal thickening of a DTS would displace the vertex of an angle inwardly and negate the claw sign (see Figure 2). That said, it is completely possible for a mass to have a DTS at one location and a claw sign at another (see Figure 2). In these cases, we suggest considering other imaging findings (e.g., neurocranium changes), breed predisposition (e.g., glioma in brachycephalic dogs), and prioritizing diseases known to be both intra‐axial and extra‐axial (e.g., histiocytic sarcoma and blastomycosis) [15, 31, 32, 33].

The DTS had almost perfect and substantial intra and interobserver agreement, respectively. These numbers compare favorably to the claw sign's intraobserver agreement (Cohen's kappa 0.72; 95% CI, 0.60–0.84) and interobserver agreement (Fleiss's kappa 0.48; 95% CI, 0.28–0.67) [5]. Because of its strong agreement, we suggest that the DTS should factor prominently in interpretation paradigms.

Several points can be made from our review of cases with disagreement. First, based on the majority opinion, a democratic committee of five image evaluators would have rated every case of meningioma positive for the DTS in every session. Such a committee would have rated every case of glioma negative for the DTS in every session with one exception. That exception (G‐9, Figure 2A,B) would have been rated negative in the first session and positive in the second session. This suggests that a collaborative and multidisciplinary team approach could be more accurate than a lone image evaluator. This is consistent with experience in human brain tumors. For example, in one study, multidisciplinary tumor board meetings resulted in a change in patient treatment in 40.5% of cases of human brain tumors. Second, some cases are inherently challenging, and applying the criterion that the DTS needs to be seen in at least two contiguous slices or two planes to be considered positive may be too constraining. Finally, if some of our image evaluators under‐read the DTS in glioma cases due to other clues causing cognitive bias (e.g., breed, adjacent hyperostosis), it is more a reflection of imperfect study design rather than ambiguity of the sign itself. Image interpreters should remember that the DTS is possible (although rare) in cases of glioma [23]. This is illustrated in Figure 2A,F. Both of these dogs with glioma were rated as positive for the DTS by several evaluators.

A study evaluating deep learning to distinguish between canine meningiomas and gliomas on MRI (without any specific reference to the DTS) reported 94% sensitivity (range, 87%–97%) and 94% specificity (range, 82%–100%) [8]. Direct comparison to our results is challenging because of differences in case selection (e.g., our requirement for glioma cases to have pial contact) and MRI field strength (3 T vs. <1 T). A deep‐learning model for detecting the DTS in human meningioma has been developed and compared to a consensus of two experienced neurologists (reference standard) [34]. The deep‐learning model was 82.22% sensitive and 17.65% specific at a low confidence score cutoff and 55.56% sensitive and 94.12% specific at a high confidence score cutoff. Together, these studies demonstrate both the potential and challenges associated with machine learning. We expect that these systems will be a valuable tool in the hands of veterinary professionals, but there is a need for transparency of the underlying methodology, training, and testing sets, as well as post‐implementation monitoring [35].

A few strengths of this study include the rigorous inclusion criteria, the number and differing backgrounds of image evaluators, and an experimental design intended to replicate a challenging yet common clinical dilemma. There are several limitations. First, there is potential for case‐selection bias as a result of the inclusion criterion of a histopathologic diagnosis. This likely favored dogs with larger masses and more advanced disease. That said, we suggest that these are the most challenging cases to interpret. Second, the study population only included meningioma and glioma. We chose to exclude other diseases (such as histiocytic sarcoma or fungal disease) because they can be intra‐axial, extra‐axial, or both [32, 33]. We designed the study to ask whether the DTS could distinguish a disease that is almost always extra‐axial (meningioma) from one that is almost always intra‐axial (glioma). It was not designed to ask whether the DTS could differentiate between different types of extra‐axial diseases. Third, contrast medium type and dosage, and the time delay from injection to imaging, were not standardized across all dogs. Four of the dogs with glioma were administered a higher dose of Gadobutrol, and none of the dogs with meningioma received the same. These differences were caused by changing hospital protocols. There was no intention to give dogs with glioma and meningioma different types and dosages of contrast medium. That said, the impact of this on our findings is thought to be low, as none of the dogs given the higher dose of Gadobutrol were positive for the DTS. It is unknown whether the variable time delay between contrast medium injection and imaging contributed to the variable image quality and disagreement. Fourth, all cases were imaged on the same 3 T MRI system using the MP‐RAGE sequence. Even though we enrolled cases over a period of 10 years, only minor changes to the pulse sequence parameters were made during that time. Our results may not translate to different image acquisition techniques. That said, several other three‐dimensional T1‐weighted gradient‐echo sequences are available from the major MRI vendors and are expected to provide similar results [36]. Finally, as previously mentioned, it is possible that evaluators allowed other features in the images to influence their evaluation. The training we provided attempted to mitigate this, as evaluators were explicitly instructed to ignore everything besides the presence or absence of the DTS.

There are several knowledge gaps to be addressed by future research. First, the histopathologic basis of the DTS in dogs remains uncertain. Second, it would be helpful to understand the agreement and accuracy of the DTS in a larger population of dogs with a variety of diseases, imaged with a variety of image acquisition techniques, and evaluated by a larger number of image evaluators. Finally, there is a need for the development and validation of hierarchical models or algorithms to aid in the diagnosis of canine brain neoplasia. We suggest that the DTS should be included in the decision tree because of its robust agreement and high sensitivity. There is also a need to discover and validate additional signs and MRI‐derived biomarkers.

In conclusion, our findings indicate that evaluators have substantial agreement on the DTS. The DTS is highly sensitive (95%) and reasonably specific (89.1%) in differentiating between dogs with meningioma and peripherally located glioma in MRI. Dogs with meningioma should be expected to have the DTS, although some cases are more challenging than others. Working as a team may improve accuracy.

## Disclosure

The EQUATOR network checklist was not used.

## Conflicts of Interest

The authors declare no conflicts of interest.

## Data Availability

The data used in the study are available from the corresponding author upon reasonable request.
